# Iron/Copper/Phosphate nanocomposite as antimicrobial, antisnail, and wheat growth-promoting agent

**DOI:** 10.1186/s12896-024-00836-7

**Published:** 2024-03-05

**Authors:** Nashwa H. Abdullah, Nouran A. Elbialy, Mohamed Abdelnaser Amer, Mostafa Kh. Gabr, Amira Salah El-Din Youssef, Mohamed H. Sharaf, M. E. Shehata, Mohamed H. Kalaba, Elham R. S. Soliman

**Affiliations:** 1 Botany and Microbiology Department, Faculty of Science, Helwan University, Cairo, Egypt; 2 Biotechnology and Biomolecular Chemistry Department, Faculty of Science, Cairo University, Giza, Egypt; 3 Zoology Department, Faculty of Science, Al-Azhar University, Cairo, Egypt; 4 Virology and Immunology Unit, Cancer Biology Department, National Cancer Institute, Cairo University, Giza, Egypt; 5 Botany and Microbiology Department, Faculty of Science, Al-Azhar University, Cairo, Egypt

**Keywords:** Antisnail, Antibacterial, Conidial germination and mycelial growth inhibition, Cytogenetic-toxicity, Drought stress, Iron/Copper/Phosphate nanocomposite

## Abstract

**Background:**

One of the current challenges is to secure wheat crop production to meet the increasing global food demand and to face the increase in its purchasing power. Therefore, the current study aimed to exploit a new synthesized nanocomposite to enhance wheat growth under both normal and drought regime. The effectiveness of this nanocomposite in improving the microbiological quality of irrigation water and inhibiting the snail’s growth was also assessed.

**Results:**

Upon the employed one-step synthesis process, a spherical Fe/Cu/P nanocomposite was obtained with a mean particle size of 4.35 ± 1.524 nm. Cu^2+^, Fe^2+^, and P^4+^ were detected in the dried nanocomposite at 14.533 ± 0.176, 5.200 ± 0.208, and 34.167 ± 0.203 mg/ml concentration, respectively. This nanocomposite was found to exert antibacterial activity against *Escherichia coli* and *Salmonella typhi*. It caused good inhibition percent against *Fusarium oxysporum* (43.5 ± 1.47%) and reduced both its germination rate and germination efficiency. The lethal concentration 50 (LC_50)_ of this nanocomposite against *Lanistes carinatus* snails was 76 ppm. The treated snails showed disturbance in their feeding habit and reached the prevention state. Significant histological changes were observed in snail digestive tract and male and female gonads. Drought stress on wheat’s growth was mitigated in response to 100 and 300 ppm treatments. An increase in all assessed growth parameters was reported, mainly in the case of 100 ppm treatment under both standard and drought regimes. Compared to control plants, this stimulative effect was accompanied by a 2.12-fold rise in mitotic index and a 3.2-fold increase in total chromosomal abnormalities.

**Conclusion:**

The finding of the current study could be employed to mitigate the effect of drought stress on wheat growth and to enhance the microbiological quality of irrigation water. This is due to the increased efficacy of the newly synthesized Fe/Cu/P nanocomposite against bacteria, fungi, and snails. This methodology exhibits potential for promoting sustainable wheat growth and water resource conservation.

## Background

Wheat (*Triticum aestivum* L.) is one of the most important economical and nutritional cereals produced for human consumption. It ranks second among the globally produced cereals with a production of 713 million tons annually [1, 2]. It ranks second among cereals globally, producing 713 million tons annually [1, 2]. It has been expected that wheat demand will increase by 70% in the coming few years due to the increased population and its increased purchasing power [3]. As a result, sustainable wheat crop production is of considerable interest, particularly given the prospect of biotic and abiotic environmental stress. Different wheat genotypes exhibited significant reductions in relative water content (RWC) and leaf characteristics when subjected to salinity and drought stress conditions. The drought and salinity stresses caused a reduction in root hydraulic conductivity (Lpr), root length (RL), root surface area (RS), root volume (RV), K + and N content in shoots and roots of three different wheat varieties. In contrast, increased levels of antioxidant enzyme activity, malondialdehyde (MDA) levels, osmotic adjustment, nonstructural carbon, and Na ^+^ content were all observed in the shoots and roots [4]. Biotic stress adversely affects the health of wheat plant and lead to a significant reduction in the yield and quality of the grains produced. It has been estimated that pests and microbial pathogens cause a 21.5% loss in the annual wheat production [5, 6]. Molluscs exert negative impacts on many crops as crop pests [7]. They infest many plants, including field crops, and cause severe damage to all plant parts [8]. *Theba pisana* could substantially reduce the percentage of wheat seedlings growth from 11.6 for the controls to 0.2 [7]. In addition to that, snails threaten human health by encouraging the spread of snail-transmitted parasites [9]. *Rhizoctonia solani* causes seedling damping-off and root rots diseases, leading to a significant loss in cereal crops [10]. Also, *Fusarium oxysporium* was reported to cause a highly degenerative growth disease in wheat seedlings [11].

On the other hand, irrigation water quality is another remarkable parameter that must be considered during the expansion of wheat crop production. As a result of water scarcity due to climate change and rapid population growth, low-quality water and treated wastewater have been used to irrigate crops in many countries [3, 12]. According to the World Health Organization report (2006), more than 10% of the world’s population consumes food irrigated by wastewater [12]. Hence, improving irrigation water quality, especially regarding microbiological contaminants, is a crucial goal. *Salmonella* spp. and *Escherichia. coli* are food-borne pathogens frequently detected in irrigation water. It has been investigated that irrigation water represents a source or a vehicle for transmitting these pathogens [13, 14].

Recently, nanoparticle-based compounds have been heavily utilized in agriculture to support plant growth and development, mitigate the stress effect through efficient nutrient utilization, and boost the production of secondary metabolites. That behavior is due to their diminutive size, vast surface area, reactivity, and high affinity for penetrating the plasma membrane [15].

Copper (Cu) and Iron (Fe) are critical micronutrients for plants and play a crucial role in controlling plant growth and development, as well as several cellular functions, including chlorophyll biosynthesis, photosynthesis, chloroplast development, and dark respiration. Additionally, phosphate represents an essential macronutrient that acts as a structural component of the nucleic acids: deoxyribonucleic acid (DNA) and ribonucleic acid (RNA), as well as a component of fatty phospholipids, which are crucial for membrane development and function. Also, it is a component of Adenosine triphosphate (ATP), which is immediately helpful for all cellular operations that require energy [16]. Copper, iron, and phosphate nanoparticles are significantly smaller than their corresponding molecules, allowing them to be rapidly absorbed by plant tissue, form more complexes with other molecules, and increase the availability of the metals to plant organs [17–20].

Concerning the antisnail activity, the toxicity of iron phosphate in the snail’s stomachs was well documented. It damages their digestive tract and causes fasting in snails in case of prolonged exposure, leading to a slow snail death [21]. Saad et al*.*, 2019 also observed a high molluscicidal activity for a copper oxide-containing nanocomposite against *Biomphalaria alexandrina* snails at low concentrations [22].

The antifungal activity of copper has been well-documented for many centuries. Indeed, copper salts have been traditionally used in many countries to control different plant pathogenic fungi. However, using copper-based nanoparticles rather than copper salts has recently gained significant interest, as the latter can be toxic in high doses. Copper-based NPs may control the release of Cu ions. Moreover, it shows a high surface area to volume ratio that enhances its antifungal activity and leads to using lower doses to yield the same effect [23]. The use of CuO nanoparticles and Cu_3_(PO_4_)_2_·3H_2_O nanosheets has shown significant effectiveness in reducing fungal diseases in watermelon (*Citrullus lanatus*) and tomato (*Solanum lycopersicum*) crops, respectively [24]. It has been investigated that the trimetallic CMC-Cu-Zn-FeMNPs can inhibit the growth of *F. oxysporum* by interfering with its ergosterol production pathway [25].

Generally, metal nanoparticles as antibacterial agents have gained significant interest due to their possible applicability as a promising alternative for antibiotics, and that limits the emergency of multidrug-resistant strains. These nanoparticles can exert their negative effect against the bacterial cell without puncturing it, thus reducing the possibilities of resistance development against NPs [26, 27]. Therefore, synthesizing NPs with antibacterial activity represents an attractive goal in the nanoscience field. Iron-based nanoparticles are one of the new materials that have gained a growing interest due to their low toxicity and eco-environmental behavior [10]. It was previously reported that the Chitosan-Fe NPs exerted antibacterial and antibiofilm activity against Gram-positive and Gram-negative bacteria. On the other hand, copper-based nanoparticles have gained significant interest as antibacterial agents. They can release copper ions and generate reactive oxygen species (ROS) extra and intracellularly, making electrostatic interaction with different biological macromolecules, deforming the cell wall, and damaging the bacterial plasma membrane [28].

Among nanomaterials, nanocomposite (NC) materials have gained significant interest due to their superior qualities, which result from incorporating multiple nanomaterial phases into a single matrix, resulting in a higher surface-to-volume ratio. Nanocomposite materials can provide regulated micronutrient delivery to plants [15]. However, to our knowledge, no studies have evaluated the applicability of Fe/Cu/P nanocomposite as an antifungal, antibacterial, antisnail, and plant-promoting agent, especially for the sustainability of wheat plant growth. So, the current study aimed to synthesis and characterize a new Fe/Cu/P nanocomposite by one step synthesis process. The synthesized nanocomposite was evaluated for its effectiveness as antisnail, antifungal and antibacterial agent to be employed for enhancing the microbiological quality of irrigation water, reducing the incidence of wheat diseases caused by fungal pathogens and restricting the spread of diseases caused by snail transmitted parasites. Additionally, the effectiveness of this nanocomposite as a nano fertilizer for enhancing wheat growth under both normal and drought stress was evaluated.

## Materials and methods

### Synthesis of Iron/copper/phosphate nanocomposite:

Iron/copper/phosphate nanocomposite (Fe/Cu/P nanocomposite) was synthesized according to Abd El-Lateef et al., 2019 method with slight modification [29]. The procedure was initiated by adding 20 ml of FeCl_3_6H_2_O “8 mM” (Loba Chemie PVT. LTD) to 20 ml of CuSO_4_.5H_2_O “4 mM” (Loba Chemie PVT. LTD) with stirring (120 rpm). During this step, 16 mM of (NH_4_)H_2_PO_4_ (Sigma-Aldrich) was added dropwise to the copper sulfate and iron chloride mixture with continuous stirring at 80°C for 6 hours until a light green milky suspension was formed. The mixture was cooled to 25°C, filtered, and rinsed with deionized water. Finally, the obtained nanocomposite precipitate was dried in oven at 90 °C for 24 hr and used for further study.

### Characterization of synthesized Fe/Cu/P nanocomposite:

The synthesized Fe/Cu/P nanocomposite was characterized utilizing different analyses. Morphologic characteristics and particle size distribution of the synthesized nanocomposite was visualized by Transmission electron microscopy (TEM). This analysis was performed using a high-resolution transmission electron microscope (HR-TEM) (JOEL model 2100, Japan) with an accelerating voltage of 8000 KV.

Phase composition and degree of crystallinity were monitored by X-ray diffraction (XRD) analysis. This test was carried out using Cu-K radiation (wavelength 1.5406°A at 40 kV and 40 mA) in the range 20°≤ 2ϴ ≤ 80° on a BRUKER diffractometer (D8 DISCOVER with DAVINCI design, Billerica, MA, USA).

The chemical composition and elemental analysis were determined by both FT-IR (spectra were measured between 400 and 4000 cm^-1^ using IR Affinity-1 spectrophotometer- Shimadzu, Japan) and X-ray Photoelectron Spectra (XPS) (ULVA-PHI INC (Japan) with AES module with Ar ion (PHI 5000 Versa probe II).

The concentration of Cu^2+,^ Fe^2+^, and PO_3_^3+^ ions were determined by ICP-OES (Ultima Expert, JOBIN YVON Technology) at the corresponding wavelength. The ion concentration was determined using ICP-OES in triplicate, and the data were expressed as mean accompanied with standard error values according to Vallapragada et al., 2011 [30].

### Biological activities of synthesized Fe/Cu/P nanocomposite

#### Antibacterial activity

The antibacterial activity of synthesized Fe/Cu/P nanocomposite has been evaluated against *Escherichia coli* ATCC 7839, *Escherichia coli* ATCC 25922, *Salmonella typhi* ATCC 6539 and *Salmonella enterica* ATCC 25566 using agar well diffusion method [31]. A warm nutrient agar medium seeded with the tested bacterium was poured on sterilized Petri dishes (9 cm diameter) and left to solidify. Using a sterile cork borer (8 mm in diameter), wells were punched on the agar plate. 100 µl from nanocomposite solution in 100 and 300 ppm concentrations were loaded on wells. Plates were incubated at 4°C overnight for diffusion of solutions and then incubated at 37 °C for 24 hours. Ciprofloxacin antibiotic discs (5 µg) were tested as a positive control, and wells inoculated with 100 µl sterile distilled water were used as a negative control. Tests were performed in a replicated manner. The antibacterial activity was assessed by observing and measuring the inhibition zone diameter (mm).

#### Antifungal activity

##### Inhibition of mycelial growth

Antifungal activity of synthesized Fe/Cu/P nanocomposite with a concentration of 100 and 300 ppm has been tested against two fungal plant pathogens, *F. oxysporum*, and *R. solani*, causing *Fusarium* wilt and *Rhizoctonia* root rot of wheat respectively. Fungal isolates were kindly provided from the Mycology lab., Faculty of Science- Helwan University. The test was carried out using the method of percent inhibition of mycelial growth (PIMG) [32, 33]. Nanocomposite has been added to warm Czapek-Dox’s agar medium, yielding 100 and 300 ppm final concentrations. The medium was poured on sterilized Petri dishes and left to be solidified. Plugs of *F. oxysporum* and *R. solani* (8 mm in diameter) were inoculated on the center of the plates. Inoculated Czapek-Dox’s agar plates with zero nanocomposite concentration have been used as a control for each fungus. Each treatment was assessed in a triplicated manner. The plates were incubated at 25 °C for 6 days. Results were taken by measuring the diameter of the fungal growth for the plates inoculated with the Fe/Cu/P nanocomposite and the control plates and calculating the PIMG as following:

$${\text{PIMG}}={\text{G}}1-{\text{G}}2/{\text{G}}1 \times 100$$

G1: Growth Diameter for control plate.

G2: Growth Diameter for plates inoculated with Fe-Cu-P nanocomposite.

##### Inhibition of spore germination

Effectiveness of Fe/Cu/P nanocomposite for suppression of *F. oxysporum* microconidia germination “the fungal isolate showed the best mycelial growth inhibition result” has been assessed. Seven-day-old *F. oxysporum* Potato dextrose agar (PDA) slants were flooded with sterile physiological saline to obtain a conidia suspension. The count of microconidia on the obtained suspension was adjusted to 15 ×10^4^ microconidia/ml using a hemocytometer under a light microscope. Fe/Cu-P nanocomposite has been added to the prepared microconidia suspension to a final concentration of 100 and 300 ppm. Treatments were incubated at 25°C in dark conditions overnight. The germination rate of microconidia was determined microscopically. Germinated and ungerminated conidia were counted under a light microscope. Tests were performed in triplicated manner and the relative conidial germination for treatments and control sample (zero Fe/Cu/P nanocomposite concentration) was calculated and represented as a percentage (%).

#### Antisnail activity

The effect of Fe/Cu/P nanocomposite on a freshwater snail (*Lanistes carinatus)* has been evaluated as follows:

##### Determination of LC_50:_

A set of *Lanistes carinatus* groups (15 snails/group) were put on separate aquaria. Different concentrations of Fe/Cu/P nanocomposite solution (20, 40, 60, 80, and 100 ppm) were added to snails’ containing aquaria. Groups with no nanocomposite treatment were used as a control. All aquaria were observed for 96 hours. During the observation process, the dead snails were counted and immediately removed. Snail death was evaluated using a dissecting needle to stimulate the foot or push the operculum in or out of the snail. The total number of dead snails was recorded daily as a mortality percentage. LC_50_ was calculated according to the formula of Behreus and Karbeur, 1953 [34]. The behavioral activity of the snails was observed after the treatment, comparable with the control group during the LC50 estimation test.

$$LC50=CM-\frac{\sum (z*d)}{m}$$

Where: CM = Maximum concentration used, z = Number of dead snails of two successive concentrations divided by 2, d = Difference between two successive concentrations, m = Number of snails in each group.

- Histological analysis of the snail:

Histological changes for organs of *L. carinatus* in treated and control snails were observed by dissecting and cutting the wanted portion of the soft tissue of the stomach, ileum, digestive gland, and gonads and fixing the tissue in Davidson fixative solution for 24 hr. Each selected sample was transferred into 70% ethanol for dehydration. Then, each sample was stained with Hematoxylin-Eosin protocol as following: all tissues were removed from the 70% ethanol solution and subjected to increasing ethanol concentrations: 80%, 90%, 95%, and absolute ethanol for 30 min per each concentration with duplicated exposure for absolute alcohol treatment. Before embedding, the dehydrated tissues were cleared in a mixture of xylene and cedarwood oil (1:1 v/v) for 24 h, then in xylene for 30 min. The cleared tissues were treated with xylene : wax (1:1 v/v). Then embedded for 4 replicates of wax for 15 minutes. Each treatment was carried out inside the oven at 60°C, then blocked. Tissues were cut by microtome at 5-8 μm thickness. Deparafinized slides were stained with Hematoxylin and Eosin stain.

#### Effect of Fe/Cu/P nanocomposite on wheat growth

*Triticum aestivum* seedlings were used to investigate the impact of produced nanocomposite on wheat plant growth. Field Crops Research Institute (FCRI), Agricultural Research Center (ARC) in Giza, Egypt (https://maps.app.goo.gl/u9Fs8mER1Fv5DUjAA) kindly provided the seeds of the “Sakha-95” wheat cultivar [35]. Growing of wheat seedlings has been carried out as following: 20 cm diameter plastic pots were filled with soil (peat moss : sand; 1:2 v/v). Each pot was seeded with 15 seed. Two concentrations of Fe/Cu/P nanocomposite have been tested (100 and 300 ppm) using a treatment of zero nanocomposite concentration as a control “received constant irrigation with tap water only “Each treatment was tested in a triplicated manner. Soil of Fe/Cu/P nanocomposite treatment groups was mixed with 500 ml of their tested concentration, and then all groups were irrigated twice weekly with a constant volume of water. After 13 days, treatments received an additional volume “150 mL” of Fe/Cu/P nanocomposite solution with the tested concentrations, whereas the control group received 150 mL of water. A second batch of the previously described groups was created and treated identically as them, except that they were exposed to drought stress by withholding the watering process for seven days at the age of 14 days. The experiment was continued with the usual watering schedule. After three weeks, seedlings of each treatment were examined, and the results were assessed.

##### Effect of Fe/Cu/P nanocomposite on the morphological parameters

The height (cm) of the plant’s shoots and roots was measured for three-week-old seedlings using a graduated ruler. The plants were harvested, and the fresh weight of the produced shoot and root biomass for each treatment was weighted. The shoot and root parts were dried in oven at 65 °C for 2 days until constant weight was obtained, and then their dry weights were determined using an electronic precision balance.

##### Cytogenetic-toxicity of Fe/Cu/P nanocomposite on wheat root apex

For recording the cytogenetic-toxicity effects of Fe/Cu/P nanocomposite on cell division and chromosomes, lateral roots of at least 5 seedlings from each treatment (0, 100, and 300 ppm concentrations) were harvested from 21 days old seedlings growing as mentioned above. The collected roots were fixed in a freshly prepared Carnoy’s fixative solution composed of absolute ethanol and glacial acetic acid (3:1) for 24 h, then kept in 70% ethanol at 4 °C until use. Feulgen-stained root tips were squashed in a drop of 45% acetic acid according to Badr et al*.*, 2018 [36] and Darlington and la Cour, 1976 [37] method, then examined using OLYMPUS CX31 microscope and photographed with ToupCam. The following formulas were used to calculate the mitotic index (MI) and total percentage of chromosomal aberration (% of CA).

$$\begin{array}{cc}MI=\frac{\mathrm{Total\ dividing\ cells }}{\mathrm{Total\ cells\ counted}}\times 100& \mathrm{\%\ of \ CA}=\frac{\mathrm{Total\ abnormal\ cells }}{\mathrm{Total\ cells\ counted}}\times 100\end{array}$$

### Statistical analysis

Results were represented as the mean value of replica ± Standard error. The comparisons between groups of treatments in the antibacterial and microconidia germination experiments were performed by one-way analysis of variance (ANOVA) followed by pairwise comparisons for grouping information using the Tukey method at 95% confidence. Means with different letters are significantly different. This analysis was carried out using the SigmaPlot statistical package version 12.5. The statistical analysis in the Cytogenetic-toxicity experiment was established by two-tailed student’s t-tests using Microsoft EXCEL at (*P 0.05, **P 0.01); *P* values reflect each treatment vs the control for the corresponding treatment.

## Results

### Characterization of synthesized Fe/Cu/P nanocomposite

**Fig. 1:**
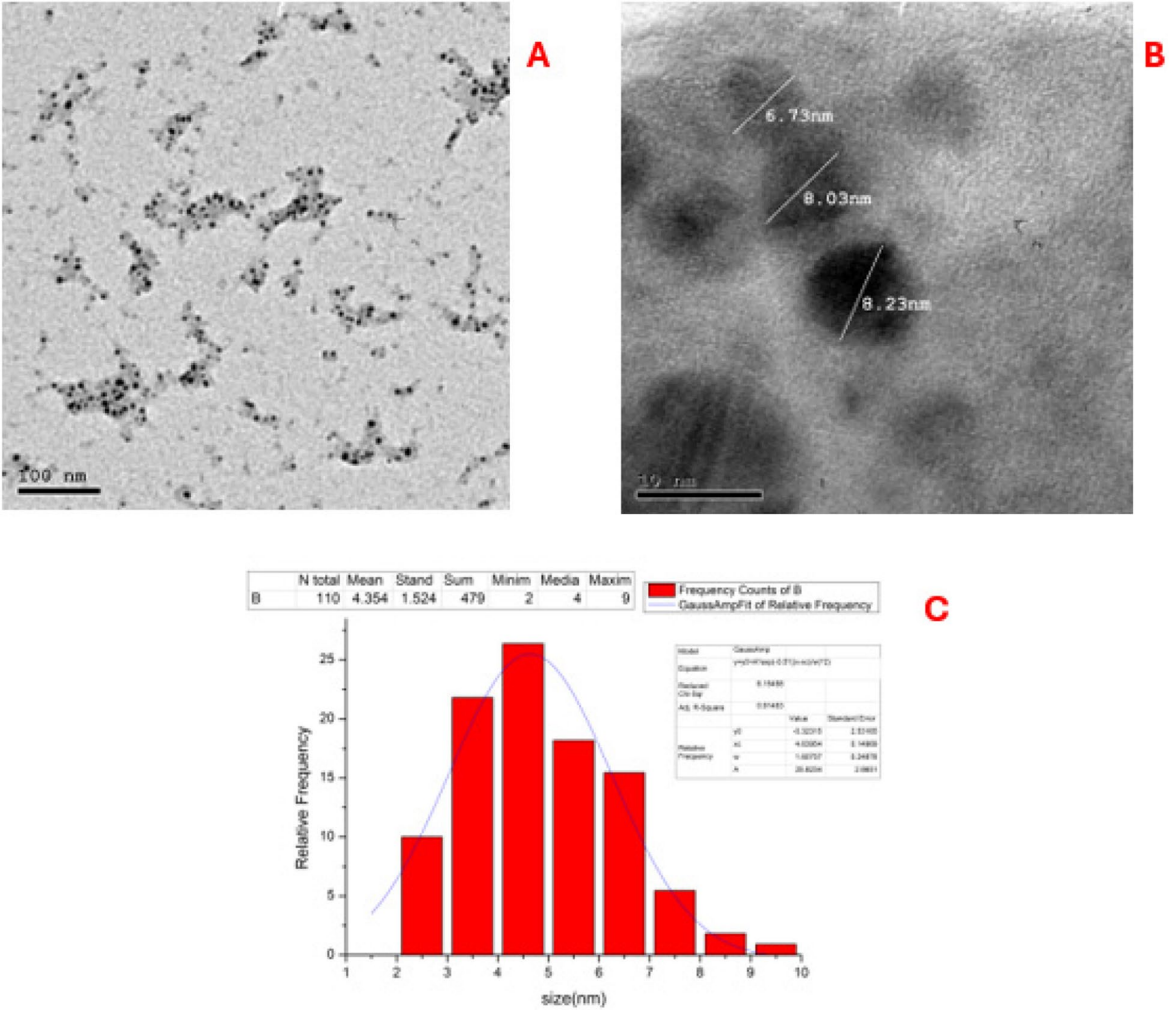
Size and morphological features of the synthesized Fe/Cu/P nanocomposite particles. TEM graph showing shape and diameter of particles at low (**A**) and high (**B**) magnification, TEM estimated particle size distribution histogram (**C**)

The HR-TEM graphs reveal that the Fe/Cu/P nanocomposite is formed in a spherical-like shape with good uniformity and without an apparent particles’ agglomeration, Fig. 1A and B. The synthesized nanocomposite showed a relatively uniform distribution in size and shape. The diameter of the nanocomposite particles was found to range between 2 and 9 nm with a mean distribution of 4.35 ± 1.524 nm as estimated by particle size distribution histogram generated by software coupled with HR-TEM, Fig. 1C.

**Fig. 2:**
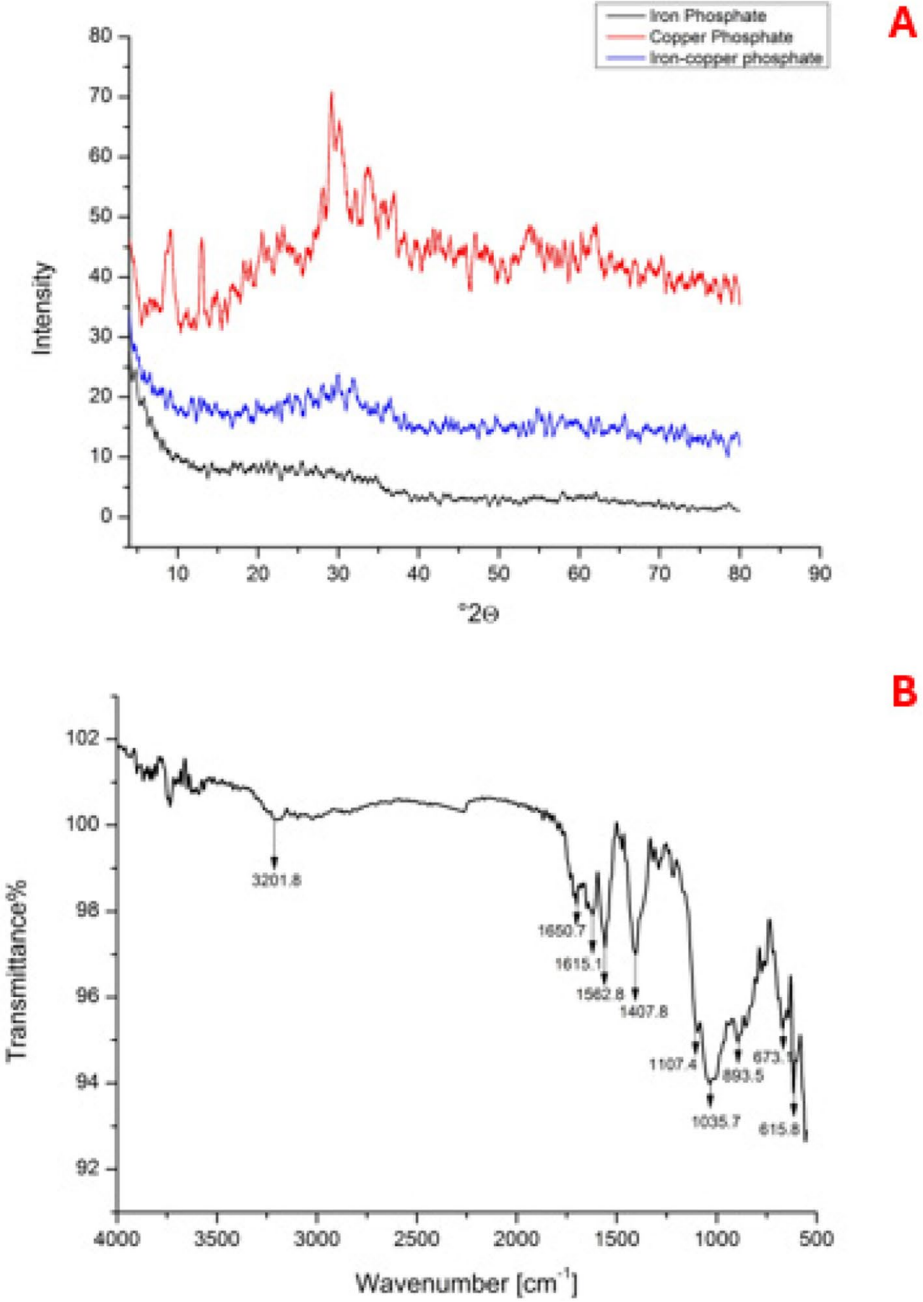
X-ray diffractograms for; **A** iron phosphate, copper phosphate and Fe/Cu/P nanocomposite, **B** FTIR analysis for the synthesized Fe/Cu/P nanocomposite

According to the obtained X-ray diffractograms, Fig. 2A, it has been estimated that all the synthesized Fe/Cu/P structures are amorphous. Nanoparticles remain amorphous despite a minor increase in phosphate structural units with the increase in the reaction time [38]. Analysis of the copper phosphate sample has revealed a 2θ peak at 29.11°, 33.93°, 37.13°, and 53.51° relative to the standard powder diffraction card of the Joint Committee on Powder Diffraction Standards (JCPDS), copper (II) phosphate file No. 80–0992 [39, 40].

The obtained FT-IR spectra of the synthesized Fe/Cu/P nanocomposite have revealed the presence of a peak at 3201.8 cm^−1^ that corresponds to the stretching vibration of O–H bonds. Other peaks have been observed at 1650.7 and 1562.8 cm^−1^ indicating the strong HOH bending of water molecules interacting with phosphate anions. Another peak at 1615.1 cm^−1^ has also been detected, representing the vibrational bending of -NH or -NH_2_ groups. The stretching vibration of P = O bonds is indicated by the peak observed at 1107.4 cm^−1^. Furthermore, the peaks observed at 673, 615, and 550 cm^−1^ correspond to the vibrations of metal–oxygen (M–O) and metal-phosphorus (M-P) bonds, Fig. 2B.

**Fig. 3:**
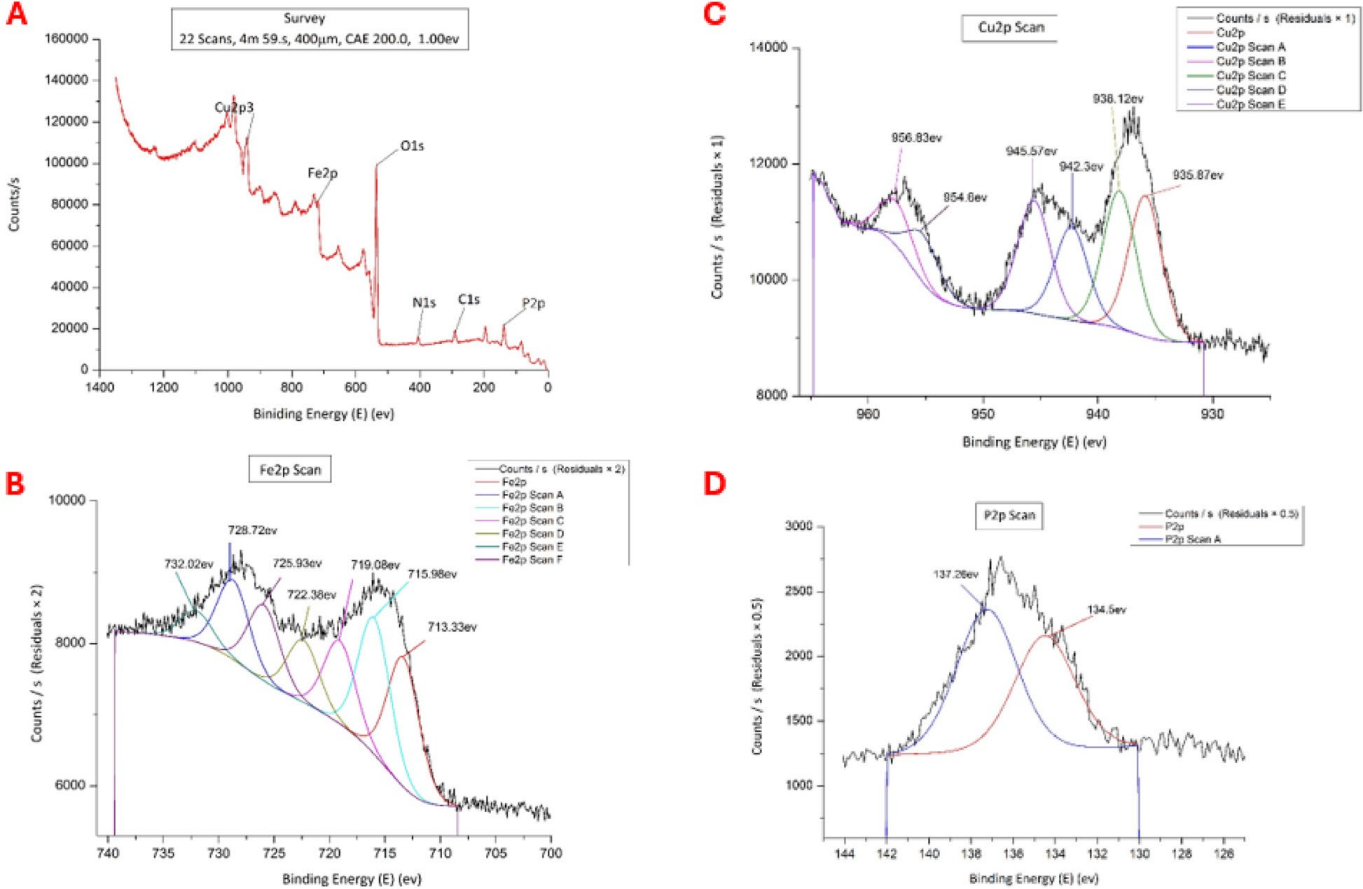
X-ray Photoelectron spectra of: **A**; Fe/Cu/P nanocomposite, **B**; Fe2P, **C**; P2P, **D**; Cu2P

X-ray Photoelectron Spectroscopy (XPS) was performed to explain the chemical composition and give information about the bonding nature of the synthesized nanocomposite. The obtained spectrum, Fig. 3A, showed that C1s (290.06 eV) and N1s peaks (405.62eV) were detected. The detailed spectra of Fe, Cu, and P region show that the binding energies of Fe2p_2/3_, Cu2p_3/2,_ and P2p_1/2_ are 714.08eV, 937.08eV and 136.68eV, respectively. The high-resolution XPS spectrum of Fe2p peak, Fig. 3B, has confirmed the presence of the Fe_3_O_4_ in the nano Fe_3_(PO_4_)_2_ due to the coexistence of Fe^3+^ and Fe^2+^ of Fe (2p_3/2_) at binding energies 715.98eV and 728.72eV, whereas the presence of Fe_2_O_3_ is attributed to the peak at binding energies 714.08 eV for Fe^3+^ oxidation state of Fe (2p_3/2_). Also, the XPS spectrum of the P2p peak, Fig. 3C, is deconvoluted into two components with binding energies 137.26eV and 134.5eV that are corresponding to P2p_1/2_ and P2p_3/2_, respectively and that indicates the presence of phosphorus element in the form of (PO_4_)^3−^. Moreover, the obtained XPS spectra have provided an overview of copper phosphate’s electronic structure and compositions. The core peak of Cu 2p, Fig. 3D, reveals the presence of two main spin–orbit splitting around 937.08eV and 957.88eV that correspond to Cu 2p_3/2_ and Cu 2p_1/2_ and that confirms the presence of Cu^2+^.

In a trail to determine the accurate concentrations of metal ions in the synthesized Fe/Cu/P nanocomposite, the content of Cu^2+^, Fe^2+^, and P^4+^ elements were estimated using emission wavelengths of 324.754, 259.94, and 213.617nm, respectively. Indeed, the dried nanoparticles have been found to contain both Cu^2+^, Fe^2+^, and P^4+^ ions at a concentration of 14.533 ± 0.176, 5.200 ± 0.208, and 34.167 ± 0.203 mg/ml, respectively.

### Assessment of biological activities

#### Antibacterial activity

**Fig. 4:**
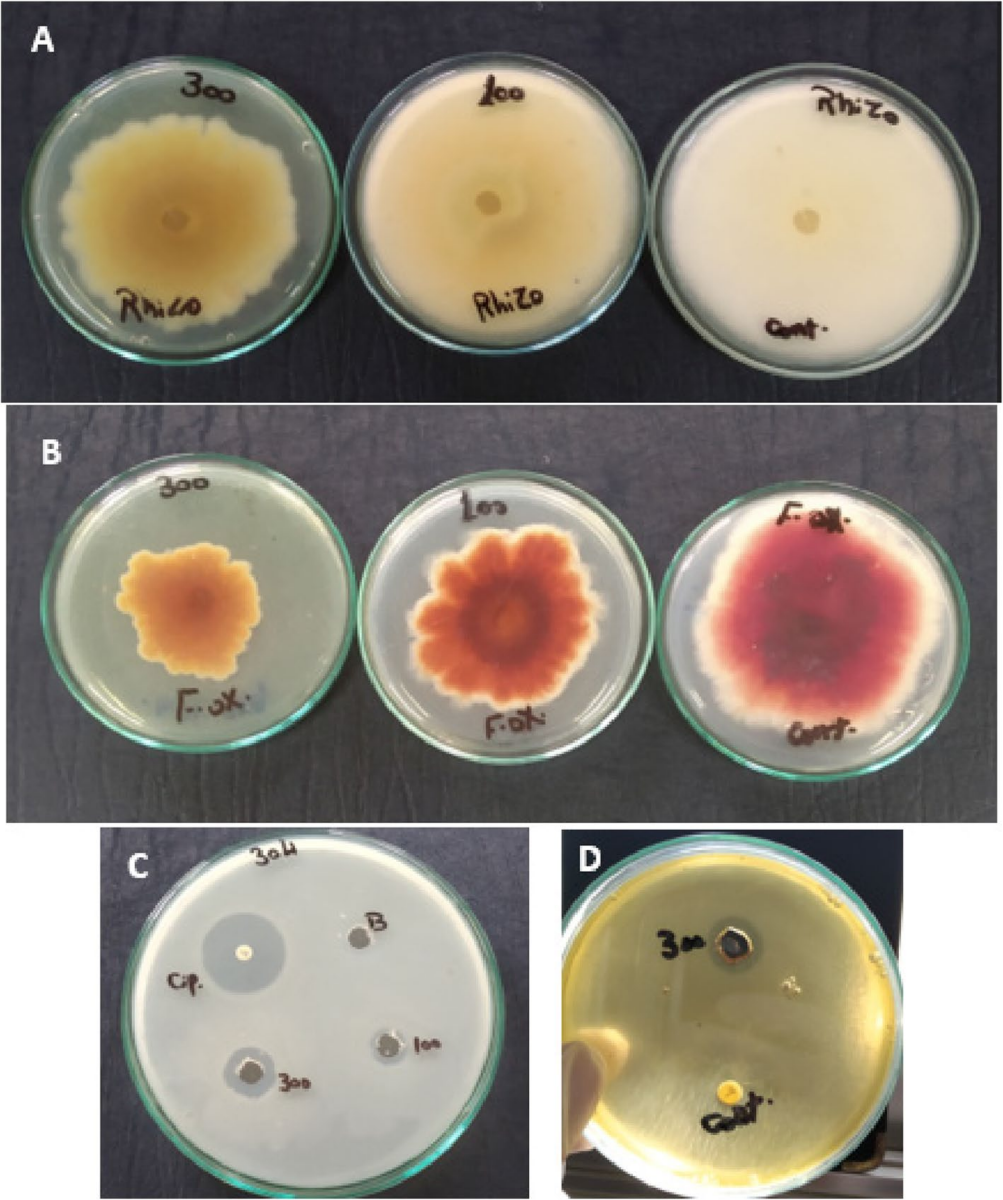
Antimicrobial activity of Fe/Cu/P nanocomposite against (**A**) *Rhizoctonia solani,* (**B**) *Fusarium oxysporum* and (**C**) *Escherichia coli* 7839, **D** *Salmonella typhi* ATCC 6539

It has been found that the investigated Fe/Cu/P nanocomposite exerts antibacterial activity against *E. coli* ATCC 7839 with both tested concentrations, Fig. 4C, and against *S. typhi* ATCC 6539 with only 300 ppm concentration, Fig. 4D. However, the tested concentrations showed no inhibitory effect against *E. coli* ATCC 25922 and *S. enterica* ATCC 25566, Table 1.

**Table 1:** Antibacterial activity of Fe/Cu/P nanocomposite. Data represent the diameter of the inhibition zone (in mm) that is formed as a result of Fe/Cu/P nanocomposite application at 100 and 300 ppm concentration in comparison to positive control (Ciprofloxacin, 5 µg) and negative control (dis. H_2_O). Means with different letters are significantly different

Treatment	Inhibition zone diameter (mm)			
	*E. coli* ATCC 7839	*E. coli* ATCC 25922	*S. typhi* ATCC 6539	*S. enterica* ATCC 25566
Fe/Cu/P nanocomposite (100 ppm)	13.75 ± 0.18^C^	0.0 ± 0.0^B^	0.0 ± 0.0^C^	0.0 ± 0.0
Fe/Cu/P nanocomposite (300 ppm)	20.25 ± 0.18^B^	0.0 ± 0.0^B^	22.0 ± 1.4^B^	0.0 ± 0.0
Ciprofloxacin (5 µg)	32.75 ± 0.53^A^	40.5 ± 0.35^A^	34.0 ± 0.0^A^	0.0 ± 0.0
Negative control (dis. H_2_O)	0.0 ± 0.0^D^	0.0 ± 0.0^B^	0.0 ± 0.0^C^	0.0 ± 0.0

The highest inhibition was exerted by ciprofloxacin (A), followed by 300 ppm nanocomposite (B) and 100 ppm nanocomposite concentrations (C, D), respectively

#### Antifungal activity

**Fig. 5:**
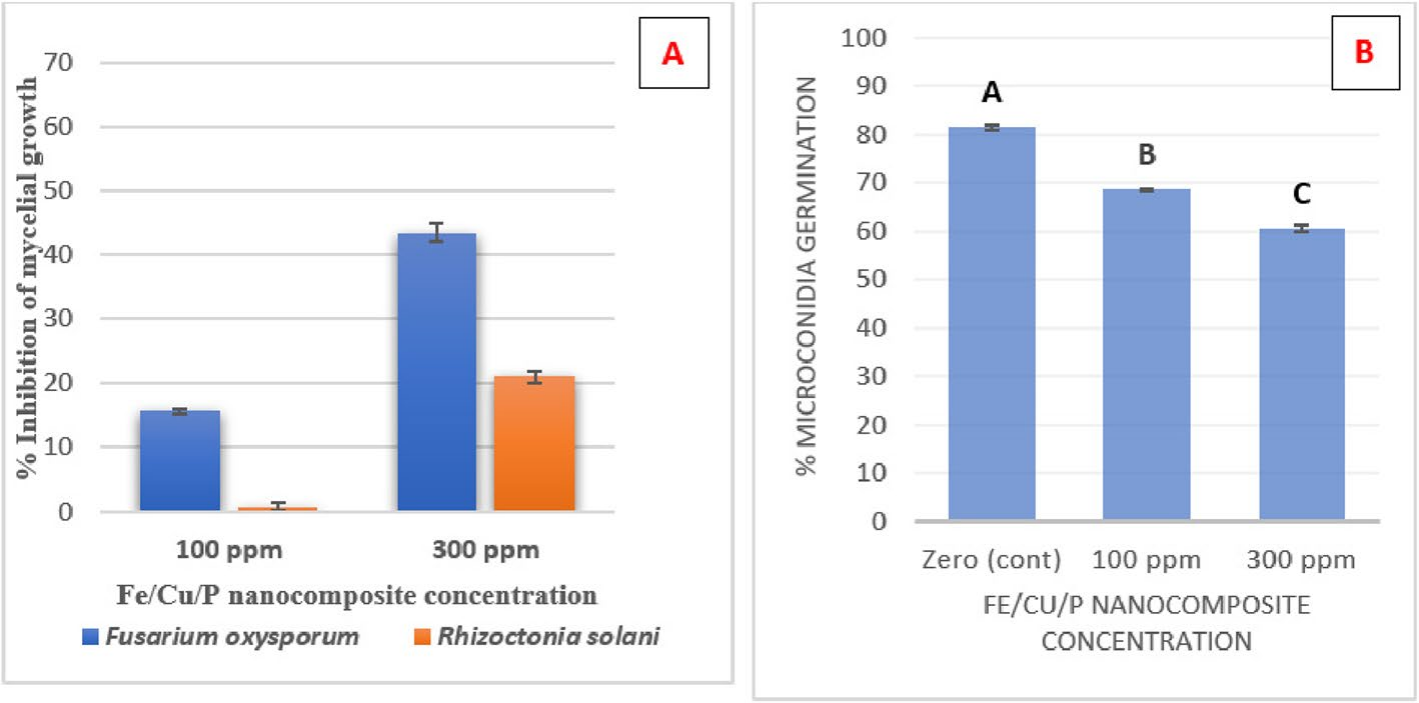
Antifungal activity of Fe/Cu/P nanocomposite: **A** Mycelial growth inhibition for *Fusarium oxysporum* and *Rhizoctonia solani*, **B** Inhibition of microconidia germination for *Fusarium oxysporum*. “Means with different letters are significantly different”

Antifungal activity of the synthesized Fe/Cu/P nanocomposite has been estimated with the same concentrations against two fungal plant pathogens affecting wheat plants (*F. oxysporum* and *R. solani*) by detecting their mycelial growth inhibition percent. Results revealed that 300 ppm concentration exerts a considerable inhibition percent against *F. oxysporum* with a value of 43.5 ± 1.47%, Figs. 4B and 5A while it inhibits the growth of *R. solani* with a value of 21.11 ±0.91%, Figs. 4A and 5A. Moreover, changes in mycelium pigmentation have been observed for both tested fungi in response to Fe/Cu/P nanocomposite treatments compared to the control, Fig. 4A and B.

Additionally, the effect of Fe/Cu/P nanocomposite on the germination of *F. oxysporum’s* microconidia (the fungus showed the best mycelial growth inhibition percent in response to Fe/Cu/P nanocomposite treatment) has been assessed by estimating the germination rate (percent of germination). Statistical analysis of the obtained results has revealed that the test was significant (*P* ≤ 0.001), where the presence of Fe/Cu/P nanocomposite has reduced the germination rate of microconidia in comparison to the control, and the differences in the mean values that have been reported among the groups of treatments are more significant than that would be expected by chance. The Pairwise multiple comparison analysis between the treatment groups has revealed that the lowest microconidia germination rate was maintained at 300 ppm concentration (60.618% ± 0.373) followed by 100 ppm concentration (68.818% ± 0.0342), and the best germination rate was recorded in the absence of Fe/Cu/P nanocomposite “control” (81.648% ± 0.309), Fig. 5B.

**Fig. 6:**
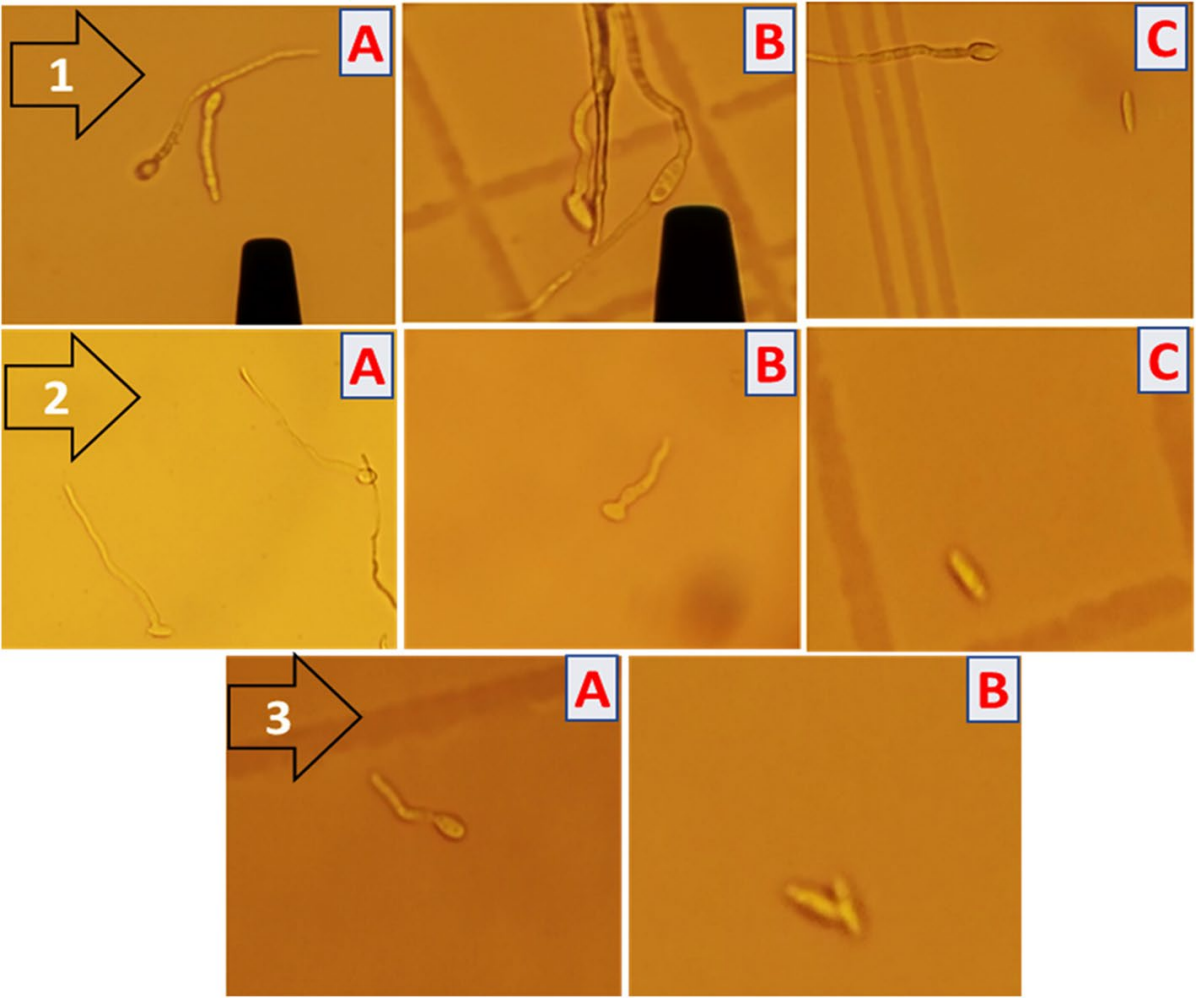
Germination *of Fusarium oxysporum*’s microconidia, [1]: control sample, (**A** germination of microconidia via a single germ pore, **B** germination of microconidia via two germ pores **C** germinated and ungerminated conidia), [2]: treatment with 100 ppm of Fe/Cu/P nanocomposite (**A** germination of microconidia via a single germ pore, **B** formation of shorter germ tube in comparison to control sample, **C** ungerminated conidium) and [3]: treatment with 300 ppm of Fe/Cu/P nanocomposite (**A** germination of microconidia via a single germ pore and the formation of shorter germ tube in comparison to control sample, **B** ungerminated conidia)

Although the germination of microconidia via two germ pores has been observed in the case of the control sample, Fig. 6–1, this germination behavior has not been recorded in the case of Fe/Cu/P nanocomposite treatments where all microconidia have germinated via a single germ pore Fig. 6–2,3. Furthermore, the formation of a longer germ tube has been detected in the case of the control sample, Fig. 6–1. These observations reveal that the presence of Fe/Cu/P nanocomposite has reduced not only the germination rate but also the germination efficiency.

#### Antisnail activity

##### Determination of LC50

Toxicity experiment was performed to determine the LC_50_ of Fe/Cu/P nanocomposite on the freshwater snail *Lanistes carinatus*. Results revealed that the LC_50_ of the investigated nanocomposite is 76 ppm.

It has been observed that the increase in nanocomposite concentration leads to a higher mortality rate among the snail population, Table 2.

**Table 2:** Estimation of Fe/Cu/P nanocomposite’s LC_50_ value for *Lanistes carinatus* snail

Trail no	Fe/Cu/P nanocomposite Conc. (ppm)	Count of snails in each group	Count of dead snails	Z	D	z X d	**LC**_**50**_
1	0	15	0	0	0	0	**76 ppm**
2	20	15	0	0	20	0	
3	40	15	1	0.5	20	10	
4	60	15	4	2.5	20	50	
5	80	15	8	6	20	120	
6	100	15	10	9	20	180	
Sum z X D						360	

During the short-term exposure of *L. carinatus* snail to different Fe/Cu/P nanocomposite concentrations (lower than the LC_50_ value), snails have shown some changes in their movement behavior, where they tend to move slowly. After 24 h exposure, the movement tends to be more slowly or stops completely, and that retards the ability of snails to move towards the food. Some snails showed another behavior response where they closed their opercula due to the addition of the investigated nanocomposite.

On the other hand, the snails show a disturbance in their feeding habit until reaching the fasting state in response to extended period of exposure.

##### Histological changes

**Fig. 7:**
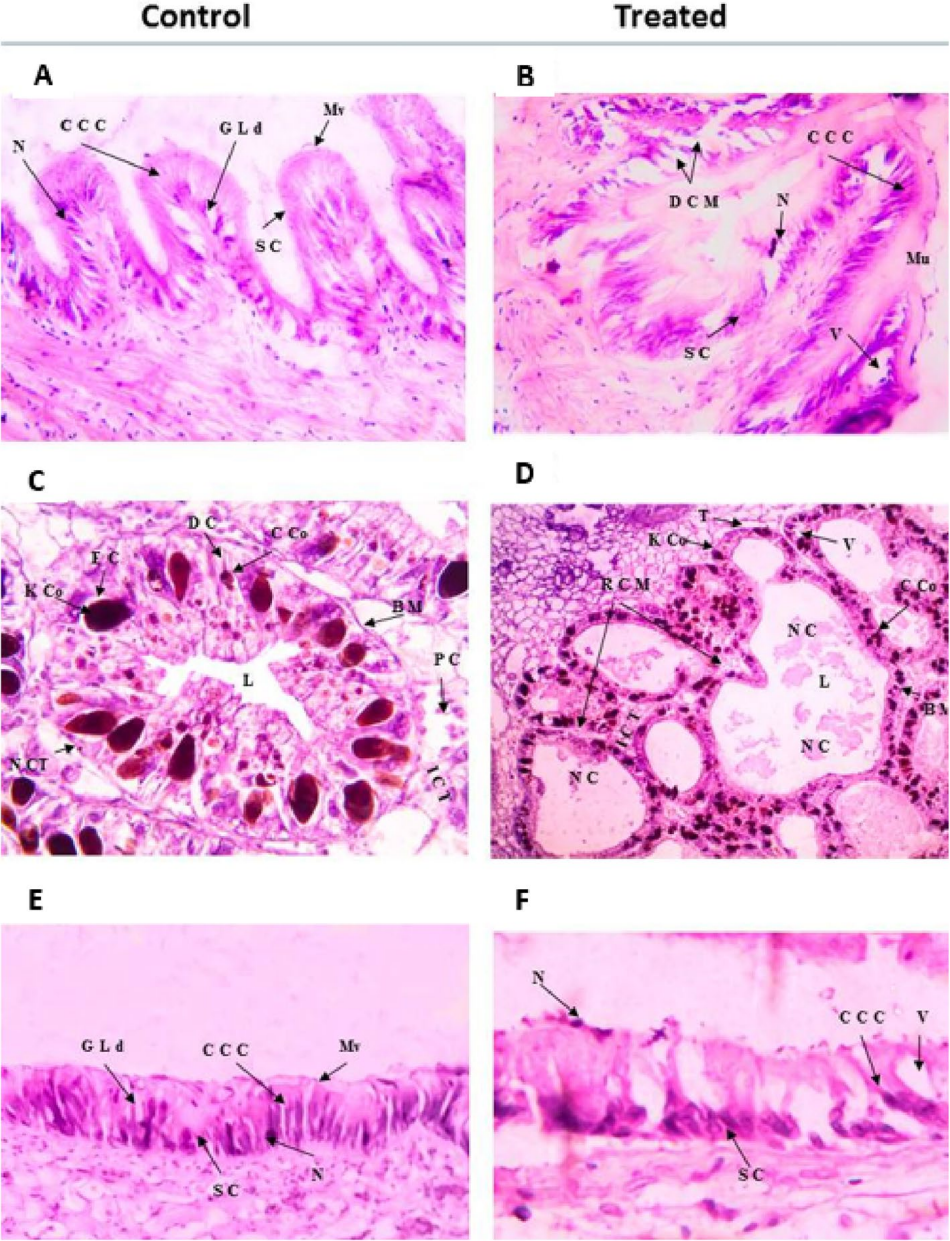
Transverse sections of *L. carinatus* organs before (control) and after exposure to the sub-lethal concentration of Fe/Cu/P nanocomposite: **A** & **B**; stomach, **C** & **D**; digestive gland and **E** & **F**; ileum. (Abbreviations: CCC: Ciliate columnar cells; GLd: Glycogen and lipid micro drops; Mv: Microvilli; N: Nucleus and SC: Secretory cells; DCM: Destroy of cell membrane; ICT: Inter tubular connective tissue; BM: Basement membrane; C Co: C corpuscle; K Co: K corpuscle; L: Lumen; NC: Necrotic cell; RCM: Ruptured cell membrane and V: Vacuoles)

**Fig. 8:**
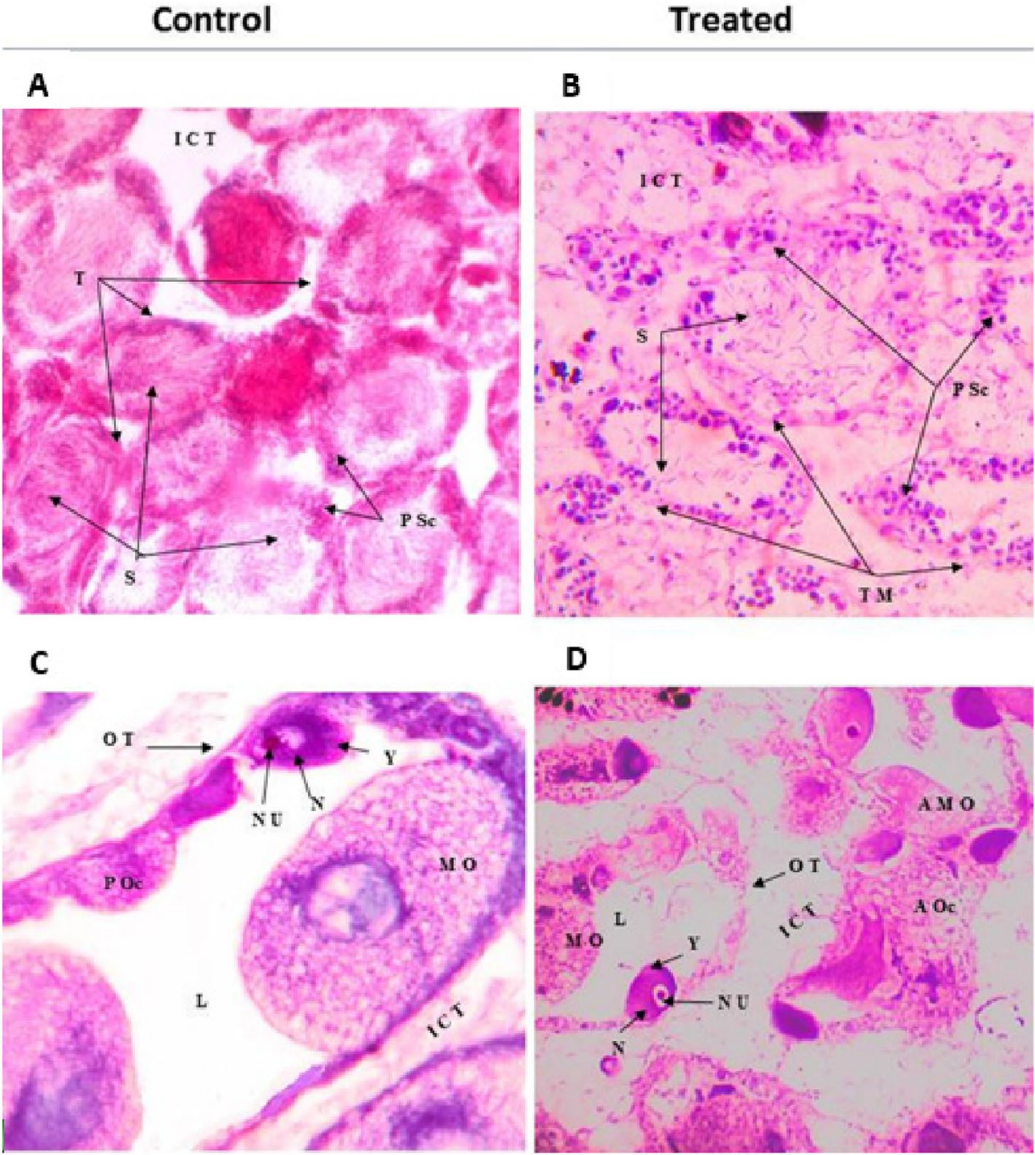
Transverse sections of *L. carinatus* gonads before (control) and after exposure to the sub-lethal concentration of Fe/Cu/P nanocomposite: **A** & **B**; testes and **C** & **D**; ovary. (Abbreviations: P Sc: Primary spermatocytes; S: Sperm and TM: Tubule membrane; AMO: Atretic mature ovum; AOc: Atretic oocyte; MO: Mature ovum; N: Nucleus; Nu: Nucleolus; OT: Ovigurous tubules and Y: Yolk)

Upon exposure to Fe/Cu/P nanocomposite, the digestive gland appears as irregularly rounded tubules, connected with each other by loose connective tissue with distinguished lumens inside them. The wall of the digestive tubules consists of two types of cells reset on the tubular basement membrane. The first cell type is the narrow elongated or columnar cells that act as digestive cells; each cell contains a basal nucleus and some rounded cellular corpuscles (C corpuscles). The second type of cell is the pyramidal cell, which has a brownish-green color and dark brown ‘club’ shaped elements known as kystic corpuscles (K corpuscles). These cells act as excretory cells, Fig. 7. After exposing *L. carinatus* snails to sub-lethal concentrations (lower than 7.6 ppm), histological changes have been observed, Figs. 7 and 8. Vacuolation was observed in the digestive gland, followed by destruction and shredding in the cell membrane, the formation of swelling, and numerous vacuoles appearing in the digestive cells with enlargement of the tubular lumen.

Moreover, the number of C and K corpuscles decreased, and many scattered cell granules were observed, Fig. 7C and D. On the other hand, the digestive tract (stomach and intestine) histology has been examined, Fig. 7A. The control group shows normal stomach epithelium formed by ciliated and unciliated columnar cells interspersed with secretory cells; both have a dense apical border of microvilli. The nuclei are found in the basal region with rough endoplasmic reticulum cisternae. Concerning the normal intestine histology, Fig. 7E it looks like the stomach; the epithelium is formed from columnar supporting cells with secretory cells, and there are microvilli on the apical surface, and most of them are ciliated. Like the stomach, many vesicles, lipid droplets, glycogen deposits, and rough endoplasmic reticulum cisternae are present in the cytoplasm of the cells.

Upon the exposure to Fe/Cu/P nanocomposite, some apparent changes have been observed, such as numerous vacuolation and swelling in epithelium columnar cells. A destroyed cell membrane has also appeared, and the apical microvilli decreased or disappeared. Concerning the stomach, cells have secreted a thick mucus layer for protection. Also, the nucleus at the basal region left the epithelium cells and became scattered.

The snail’s ovary in the control group shows many ovarian tubules, which are somewhat rounded or oval in outline. Each tubule cross section has a thin wall composed of an outer coat of connective tissue surrounding a germinal epithelium and is formed of flattened or somewhat cubical cells with large oval nuclei, which give rise to large egg cells. Each mature egg cell contains a large nucleus with a conspicuous nucleolus embedded in a highly reticular cytoplasm. The tubules’ lumen is relatively empty when the snail is not producing eggs. Before laying eggs, the lumen is filled with large-sized mature eggs. After exposing the snails to the sub-lethal concentration of the investigated nanocomposite, the snails’ ovaries showed fewer eggs and started degeneration in some oocytes. Atretic tubules and oocytes inside it also appeared, and the degeneration in the ova and the debris of the mature eggs were observed in the lumen of the ovarian tubules. Moreover, an increase in the connective tissue between tubules and an enlargement in the tubular lumen was observed, Fig. 8C and D. On the other hand, normal testis in the control group, consisting of a flattened tubular gland matrix, was observed. Each tubule contained numerous numbers of inactive sperms, which had enlarged heads and long tails. These flattened tubules are separated from each other by a thin layer of connective tissue. Some primary spermatocytes were observed as aggregated cells on the internal side of the tubular wall. Some dramatic histological changes have evolved after exposure to the investigated nanocomposite, such as increased inter-tubular connective tissue and destruction in the seminiferous tubular fence. The primary spermatocytes were decreased, and they left the tubular walls, then scattered inside the tubular lumen. Also, sperms were significantly reduced in numbers, split from the tubular wall, and irregularly scattered and dispersed in the lumen Fig. 8A and B.

#### Effect of Fe/Cu/P nanocomposite on wheat growth

**Fig. 9:**
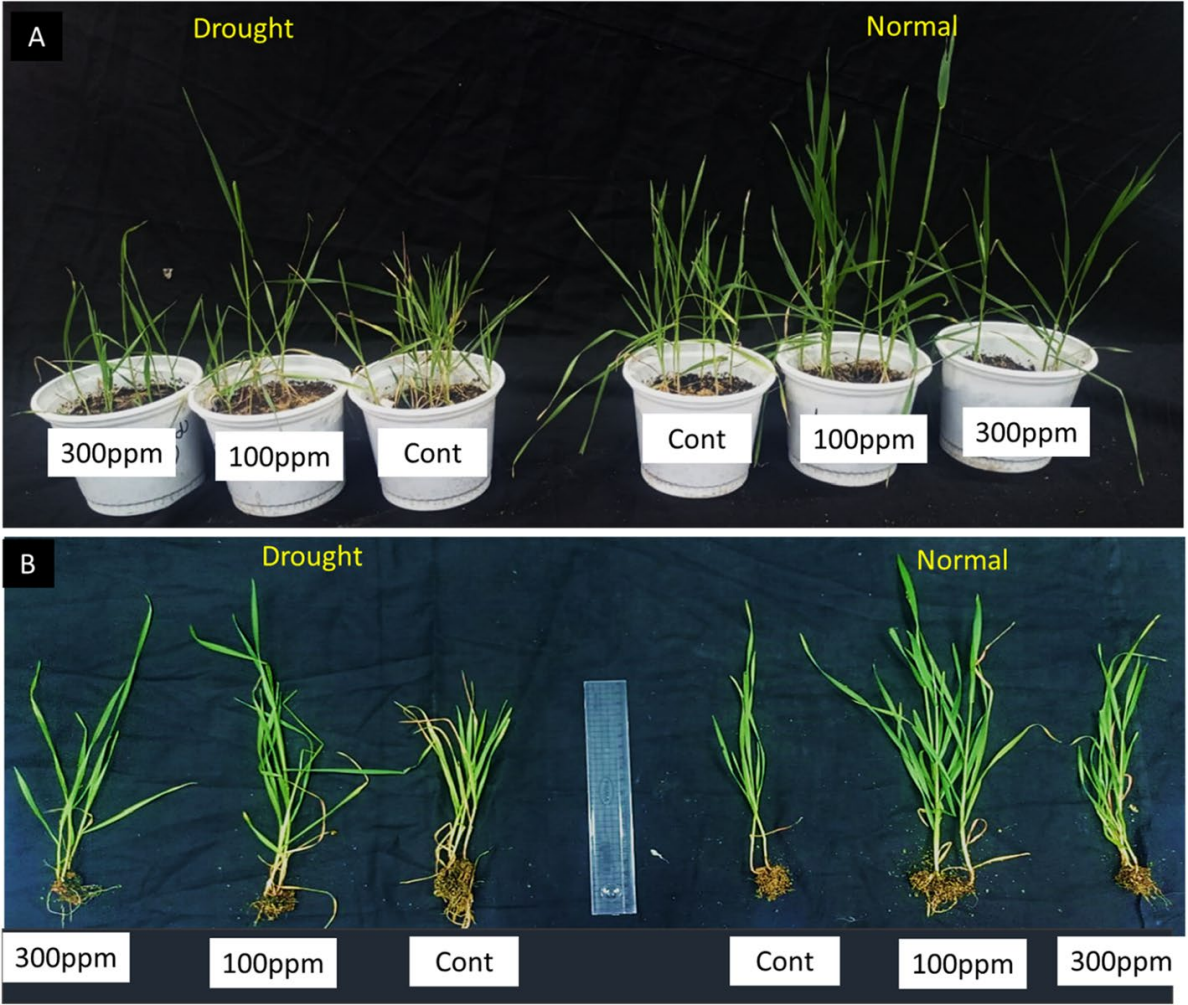
Effect of 100 and 300 ppm Fe/Cu/P nanocomposite concentrations on wheat growth under both control (normal watering regime) and drought stress. **A** shoot growth of 50 days old wheat plants. **B** vegetative growth of 50 days old wheat plants showing shoot and root growth

Although all assessed growth parameters were lowered in the case of drought-stressed plants, this effect was mitigated in plants treated with Fe/Cu/P nanocomposite. It has been found that the applied concentrations have promoted wheat growth under both drought stress and normal watering program conditions. Moreover, after only two months from sowing, wheat plants treated with 100 ppm have developed spikes in comparison to control and 300 ppm treated plants, Fig. 9.

**Fig. 10:**
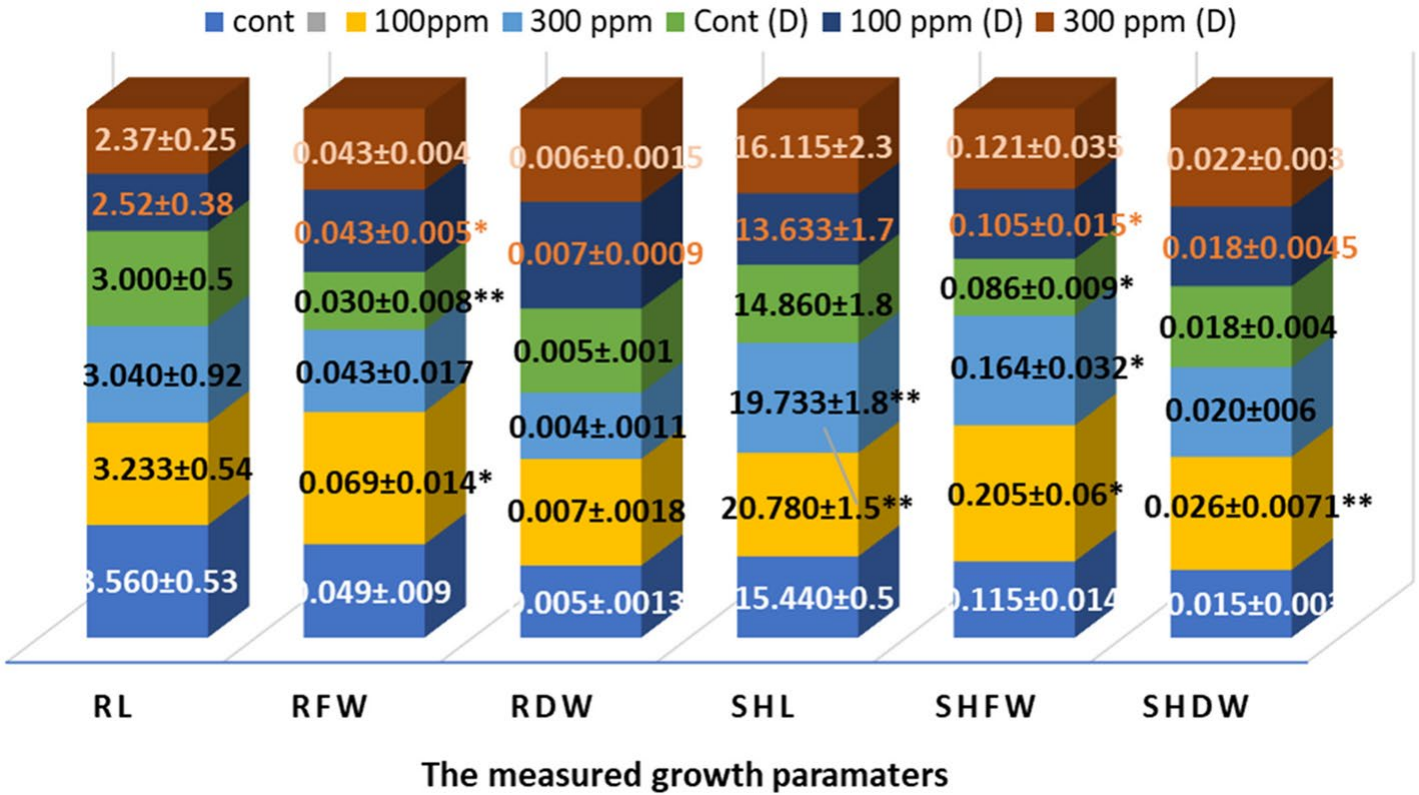
Effect of 100 and 300 ppm Fe/Cu/P nanocomposite concentrations on wheat growth parameters “Control and nanocomposite treatments under drought stress (D)”: (RL); root length, (RFW); root fresh weight, (RDW); root dry weight, (SHL); shoot length, (SFW); shoot fresh weight, and (SDW); shoot dry weight. The letter “(D)” is utilized to denote the drought in the key bar. (*P < 0.05, ** *P* < 0.01); *P* values represent each treatment versus the corresponding control. (± SD) represents standard deviations

The root measured parameters, especially fresh and dry weights, were significantly improved by Fe/Cu/P nanocomposite application, especially with 100 ppm concentration, yielding a 40.8 and 40 % increase compared to the control plants. On the other hand, all shoot-measured parameters were improved by both 100 and 300 ppm concentration, yielding a significant increase in shoot length and fresh and dry shoots’ weight with a value of 34.6, 78.2, and 73.3 %, respectively, in the case of 100 ppm concentration, in comparison to control, Fig. 10.

##### Cytogenetic-toxicity of Fe/Cu/P nanocomposite on wheat root apex

**Fig. 11:**
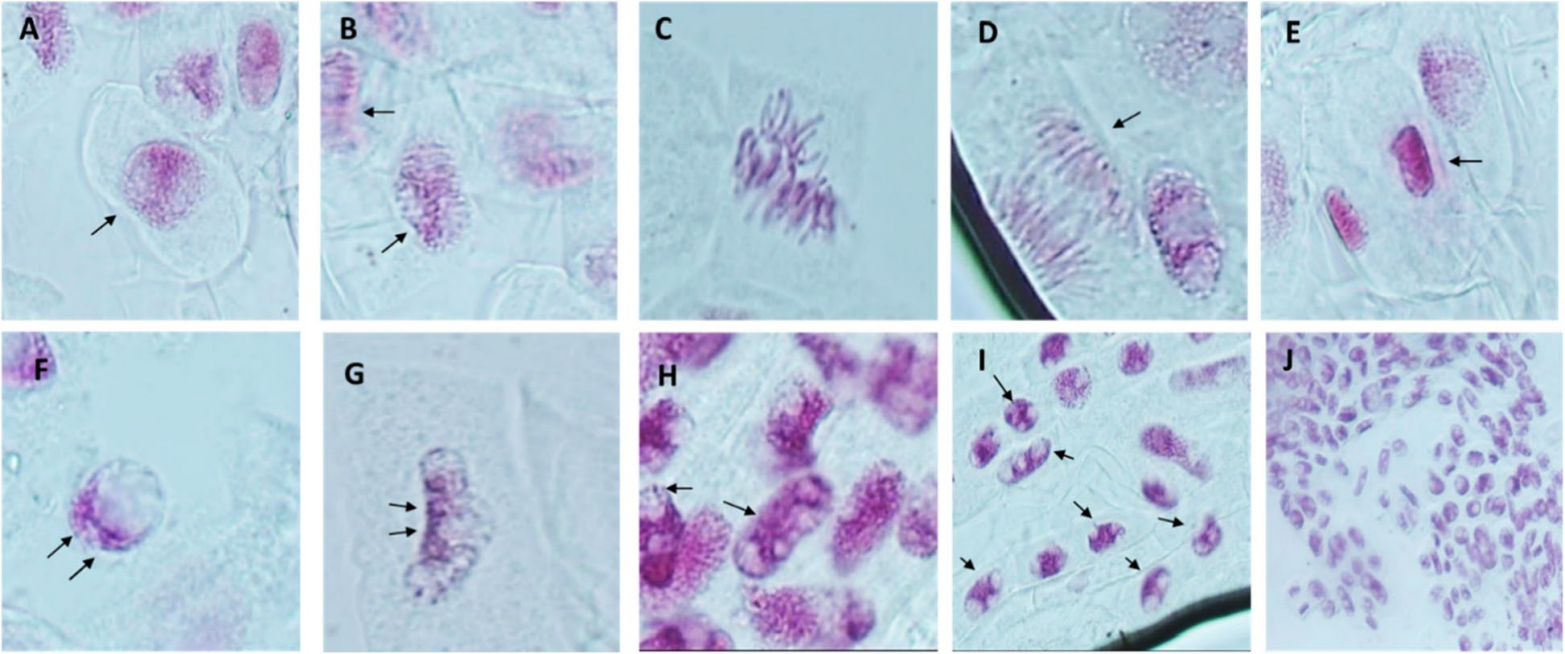
Different normal mitotic phases and examples of some chromosomal aberration observed in root apices of wheat plants treated with (0, 100, 300 ppm) of Fe/Cu/P nanocomposite: **A**; shows normal interphase stained cell, **B**-**E**; show all normal mitotic phases as follows, prophase, metaphase, anaphase and telophase, **F**-**G**; show heterochromatinized cells, **H**-**I**; show multinucleoli cells and (**J**); shows example of the examined fields

Feulgen-stained wheat root apices of all examined plants in the control group have displayed a typical pattern of cell division with 1.5% chromosomal abnormalities. Compared to control plants, the Mitotic index (MI) of plants treated with 100 ppm Fe/Cu/P nanocomposite concentration increased by 2.12-fold. This increased MI was accompanied by a 3.2-fold increase in overall chromosomal abnormality compared to control plants. The MI of 300 ppm-treated plants was 4.1±1, whereas that of untreated plants was 3.9±0.2. Most chromosomal abnormalities detected in Fe/Cu/P nanocomposite-treated plants were multinucleoli and Heterochromatinization, Fig. 11 and Table 3.

**Table 3:** The average percent of mitotic indices (MI) and chromosomal aberrations (CA) observed in root apices of wheat plants treated with (0, 100, 300 ppm) of Fe/Cu/P nanocomposite

Treatment	Control	100 ppm	300 ppm
% MI	3.9 ± 0.2	8.3 ± 0.66^*^	4.0 ± 1
% CA	1.5 ± 0.2	4.8 ± 0.3^*^	1.2 ± 0.2

^*^P < 0.05; *P* values represent each treatment versus the corresponding control. (± SD) represents standard deviations

## Discussion

Wheat represents a valuable edible crop. It is an essential supply of carbohydrates and proteins and provides the caloric demand of 1/3rd of the world’s population [3, 41]. Hence, this study aimed to provide an ecofriendly approach to improve wheat plant growth and enhance its protection against biotic and abiotic stress in a trial to secure this essential food resource and meet the increasing food demand. Additionally, this study has assessed the effectiveness of this approach for improving the microbiological quality of irrigation water that represents a source/vehicle for transmission of food-borne pathogens, mainly due to the water scarcity and the use of waste and treated water in agriculture practice in some countries.

Synthesizing nanomaterials has been exploited as a promising eco-friendly approach that resolves many agricultural issues. Nanomaterials have been successfully employed as plant growth promotors by enhancing nutrient uptake. Also, they have been exploited as effective inhibitors for many plant pathogens [1, 2].

A nanocomposite is a multiphase material in which one of the phases has one, two, or three dimensions of less than 100 nm, or the composite phases have nanoscale distances [42]. Among nanomaterials, nanocomposite (NC) materials have gained significant interest due to their superior qualities, which result from incorporating multiple nanomaterial phases into a single matrix, resulting in a higher surface-to-volume ratio [15].

This study synthesized a Fe/Cu/P nanocomposite via a one-step approach to provide a simple, fast, and cost-effective synthesis method. Particles of the obtained nanocomposite showed nearly identical shapes and good size distribution with a mean particle size of 4.35 nm as revealed by TEM analysis. These findings suggest that the employed synthesis method has efficiently regulated the growth and development of the produced particles and represents a promising synthesis method for Fe/Cu/P nanocomposite.

X-ray diffractograms reveal that none of the synthesized phosphates have a crystalline structure. Thus, they are amorphous, meaning the phosphates’ atoms are irregularly arranged. The nanoparticles remain amorphous despite a slight increase in the number of phosphate structural units with the increase in the reaction time. This suggests that the increase in structural units is insufficient to cause crystallization. The orthophosphate portion of the iron phosphate sample crystallizes poorly. During the analysis of the copper phosphate sample, distinct peaks were observed at the 2 angles of 29.1162°, 33.9320°, 37.1386°, and 53.5124°. These peaks are compared to the standard particle diffraction card from the Joint Committee on Particle Diffraction Standards (JCPDS) for Copper (II) Phosphate, file number 80-0907 [38, 39, 43].

Functional groups that contribute to the nanocomposite formation have been monitored by the FTIR analysis. The results revealed a peak at 3201.8 cm^−1^ that is correlated to the stretching vibration of O–H bonds and indicates the presence of hydroxyl groups or water molecules within the nanocomposite. That may be related to the water film on the particles’ surface or the incorporation of hydroxyl groups through surface functionalization or the synthesis process. Similarly, the peak observed at 1615.1 cm^−1^, which corresponds to the bending vibration of -NH or -NH_2_ groups, represents evidence for the presence of amine or amino groups in the synthesized nanocomposite. These groups are likely due to the use of (NH_4_)H_2_PO_4_ in the synthesis process.

The peak at 1650 cm^−1^ may correspond to the stretching vibration of carbonyl (C = O) groups, while 1407 cm^−1^ may be related to the deformation vibration of -CH_3_ or -CH_2_ groups. These might be associated with the presence of adsorbed species or surface contaminants. Appearance of a peak at 1107.4 cm^−1^ suggests the presence of phosphate groups or other phosphorus-containing compounds. These components may originate from the precursor materials employed in synthesizing the nanocomposite. Additionally, the peaks observed at 673, 615, and 550 cm^−1^, which correspond to metal–oxygen (M–O) and metal-phosphorus (M-P) bond vibrations represent a further support for the presence of metal–oxygen and metal-phosphorus interactions within the nanocomposite [38, 39, 44].

The XPS analysis on the survey spectrum revealed two prominent peaks at 290.06 eV and 405.62 eV. The peak at 290.06 eV is attributed to residual (C1s) impurities that have adsorbed on the sample’s surface when it was exposed to air, while the peak at 405.62 eV is attributed to N1s, which may be associated with the use of (NH_4_)H_2_PO_4_ during the preparation. The binding energy at 715.98 eV corresponds to Fe^3+^ (indicating the presence of Fe_2_O_3_), while 728.72 eV corresponds to Fe^2+^ (suggesting the presence of Fe_3_(PO_4_)_2_, a form of nanostructured iron phosphate) [45–47]. The observed Cu2p3/2 and Cu2p1/2 binding energies (937.08 eV and 957.88 eV) correspond to the Cu2p3/2 and Cu2p1/2 core-level electrons, respectively. The presence of two distinct peaks suggests the presence of a Cu^2+^ oxidation state in the sample, providing information about the electronic structure and composition of copper phosphate. Concerning the P2p1/2 and P2p3/2 binding energies (137.26 eV and 134.5 eV), these binding energies correspond to the P2p1/2 and P2p3/2 core-level electrons, respectively. The presence of two distinct components suggests the presence of phosphorus in the form of (PO_4_)^3−^, indicating the presence of phosphate in the sample [48, 49].

The elemental contents of the nanocomposite were detected directly by employing the ICP-OES analysis. The reported results confirm the presence of Cu^2+^, Fe^2+^, and P^4+^ ions with a concentration of 14.533 ± 0.176, 5.200 ± 0.208, and 34.167 ± 0.203 mg/ml respectively, in the dried nanocomposite. These results provide quantitative information about the concentrations of these ions, helping in a complete understanding of the nanocomposite’s elemental composition.

The antibacterial activity of the synthesized Fe/Cu/P nanocomposite has been evaluated against three members of the family Enterobacteriaceae (*E. coli*, *S. enterica*, and *S. typhi*) that have been commonly detected in irrigation water and represent a public health issue. It has been found that the investigated nanocomposite has exerted antibacterial activity against *E. coli* ATTC 7839 with 100 and 300 ppm concentrations and against *Salmonella typhi* ATTC 6539 with 300 ppm concentration.

Although few studies have been conducted to evaluate the antimicrobial activities of iron or copper phosphate nanoparticles, none have considered the antimicrobial activity of Fe/Cu/P nanocomposite. Results reveal that Fe/Cu/P nanocomposites are one of the iron-based nanomaterials that shows an enhanced antibacterial behavior compared to iron nanoparticles.

Elsawy et al., 2021 have reported antibacterial activity for synthesized amorphous and crystalline iron phosphate nanoparticles against *E. coli*. The effectiveness of these nanoparticles against Gram-negative bacteria was higher than that of Gram-positive, which reflects their selective inhibition of Gram-negative bacteria [26]. In agreement, Pinheiro et al*.*, 2023 [50] have reported that the Cu nanoparticles show higher antimicrobial selectivity against gram-negative bacteria, which can be explained by the higher affinity of their positively charged ions towards these bacteria’s negatively charged cell walls. However, iron nanoparticles synthesized by Sandupatla et al., 2021 using *Passiflora edulis* have recorded no antibacterial activity against *E. coli* [51].

On the other hand, the effectiveness of the synthesized Fe/Cu/P nanocomposite against *F. oxysporum* and *R. solani* has been evaluated by estimating their mycelial growth inhibition percent. Fe/Cu/P nanocomposite at 300 ppm concentration has exerted a good inhibition percent against *F. oxysporum* (43.5 ± 1.47%) and inhibited the growth of *R. solani* with a value of 21.11 ± 0.91%. On the other hand, a change in the mycelial growth pigmentation has been observed in the case of Fe/Cu/P nanocomposite treatment for both fungi compared to their control sample. The observed pigmentation changes can reflect the elevated oxidative stress generated by the action of Fe/Cu/P nanocomposite, as it has been reported that fungi can produce some pigments, such as melanin and carotenoids, in response to oxidative stress. Indeed, it has been documented that melanin pigment can enhance the survival of fungi under environmental stress. Also, carotenoids were found to protect fungi against the oxidative stress [23]. Thus, such color change can reveal the exposure of the tested fungi to elevated oxidative stress in the case of Fe/Cu/P nanocomposite treatments.

To the best of our knowledge, this is the first published data about the antifungal activity of Fe/Cu/P nanocomposite against *F. oxysporum* and *R.a solani*. Thakur et al*.,* 2020 evaluated the antifungal activity of barium ferrite nanoparticles against *F. oxysoporum*. These nanoparticles have yielded 61.11% and 57.78% mycelial growth inhibition for *F. oxysoporum* when tested with 300 and 200 ppm concentrations, respectively [52]. However, Hermida-Montero et al*.,* 2019 have tested the effectiveness of synthesized copper and copper oxide nanoparticles against *F. oxysporum*. They have reported deficient antifungal activity against *F. oxysporum*, yielding less than 16% radial growth inhibition at 250 ppm concentration [23]. On the other hand, Sandupatla et al*.,* 2021 have detected good activity against *R. solani* for photosynthesized Fe nanoparticles using *Passiflora edulis* [51]. The mycelial growth of *R. solani* was inhibited with 30.86, 49.38, and 53.08 percent when treated with 100 ppm concentration of TiO_2_, ZnO, and SiO_2_ nanoparticles, respectively [53].

Metallic nanoparticles can affect cell membrane proteins and oligosaccharides through oxidative damage via Fenton reaction [51]. The generated ROS can exert oxidative stress, stimulating proteolysis on the microbial cell and uncontrolled cell destruction [53]. Indeed, many studies have revealed that iron can be involved in the generation of ROS, such as hydroxyl (^⋅^OH) and superoxide (O_2_^−^) radicals, which can interfere with the electron transport process during the oxidation of nicotinamide adenine dinucleotide in the bacterial cell [54]. Also, the produced ROS can interfere with the cell wall, cell membrane, DNA, RNA, and other cellular components, causing inhibition of fungal growth [52]. On the other hand, the penetration of nanoparticles through the plasma membrane can lead to mechanical damage and microbial cell death [51].

As conidia germination is an essential stage in the fungal infection process, the germination behavior of *F. oxysporum* microconidia in the presence and absence of Fe/Cu/P nanocomposite has been monitored. The germination rate of *F. oxysporum* microconidia was decreased in the presence of Fe/Cu/P nanocomposite. The lowest microconidia germination rate was maintained at 300 ppm concentration (60.618% ± 0.373) compared to the control (81.648% ± 0.309). Moreover, better germination efficiency has been observed in the absence of Fe/Cu/P nanocomposite, where a microconidial germination via two germ pores and more extended germ tube formation was detected in absence of Fe/Cu/P nanocomposite. In a previous study the treatment of *F. oxysporum* with 0.5 mM of hydrogen peroxide was found to suppress the conidial germination to 64.6% compared to the control case (89.5%) [55].

Numerous nanoparticles have shown significant results as molluscicides [22, 56, 57]. in accordance, the current results showed that the investigated nanocomposite exerts significant damage in the digestive gland of (*Lanistes carinatus*). Destruction and shredding in cell membrane, swelling, and numerous vacuoles in the digestive cells with enlarged lumen have been detected. This damaging effect can be explained by the potential of nanoparticles to penetrate the biological systems and permeate across cell membranes more efficiently than the larger particles [58, 59]. Copper has been detected to exert similar damaging effects in *Marisa cornuarietis* [60]. Concerning the impact of the investigated nanocomposite on snail’s gonads, male and female gonads have shown dramatic effects. The reported results are consistent with the findings detected by El-Khayat et al*.*, 2018 [61] and Saad et al*.*, 2019 [22] for *Biomphalaria alexandrina* snail when exposed to CuO nanoparticles. Most studies that have evaluated the effect of nanoparticles on snails have targeted their digestive gland as it functions similarly to the liver in higher animals; it removes xenobiotics from their bodies [62]. In current study, the application of nanocomposite has caused snail cell vacuolation and swelling of epithelium columnar cells, destruction of the cell membrane, disappearance of the apical microvilli, and scattering of the cell nucleus in the digestive tract tissues (stomach and intestine).

In a trial to evaluate the effect of the synthesized Fe/Cu/P nanocomposite on the growth and development of wheat plants, the mitotic index was utilized to indicate cell activity and proliferation [36]. The roots and shoots measured metrics were improved significantly due to Fe/Cu/P nanocomposite treatment, particularly at 100 ppm, which was associated with a 2.13-fold rise in MI. Due to their nano size, Fe/Cu/P nanocomposite can be quickly translocated to roots and shoots, which may account for the beneficial effects of their application. Increased phosphorus and iron availability in plant leaves may create Fe-phosphate species, which promote plant growth and development. The 100 ppm Fe/Cu/P nanocomposite concentration treatment has resulted in an apparent stimulative effect. Only two months old plants in this treatment have developed spikes. This response can be explained by the fast accumulation of the needed micronutrients, copper, iron, and phosphate, which stimulates the development of the plant and becomes able to initiate the reproductive phase [19]. Elevated rates of mitotic index have been recorded. This may reflect an increase in photosynthesis and respiration rates compared to the control population. This is congruent with the fact that the application of Fe_3_O_4_ nanoparticles to *Triticum aestivum*, CuO nanoparticles to *Brassica juncea*, and CaPO_4_ nanoparticles to various plant species resulted in increased photosynthesis and respiration rates, as well as enhanced leaf and root growth [15, 17, 63]. Drought is the most significant abiotic stress encountered by plants that limit crop yield and threaten food safety. Nanoparticles have been reported to mitigate the loss in carbon assimilation caused by drought by enhancing photosynthetic activity.

Additionally, they induce enhanced root development, upregulation of aquaporins, alteration of intracellular water metabolism, accumulation of compatible solutes, and ion homeostasis [64]. Applying Fe/Cu/P nanocomposite mitigates the effect of drought stress at both concentrations, but the effect was more pronounced at 100 ppm. A similar response was observed when drought stressed *Setaria italica* plants were treated with Fe nanoparticles, where they not only acted as a supply of iron as an essential nutrient but also assisted the plant in overcoming drought stress. The stimulative increase in chlorophyll and soluble sugar content and the overall increase in plant growth suggest that the iron absorbed by the plants was utilized to produce photo-assimilating molecules [65].

On the other hand, interestingly, the assessment of the cytogenetic-toxic effect of the Fe/Cu/P nanocomposite on wheat plants revealed an increase in mitotic activity in plants treated with a concentration of 100 ppm. In a few instances, the MI of cells treated with 300 ppm was equivalent to or higher than that of the control group. This observation is likely related to changes in the cell cycle of root meristems. In previous studies, it has been found that the mitotic index was lowered, and chromosomal abnormalities such as chromosome breakage, asynchronous division, advanced chromosomes, micronucleus, and genetic material loss were increased in *P. vulgaris* exposed to 300 ppm CuO nanoparticles [66].

Abdelsalam et al*.*, 2022 have estimated the range of chromosomal aberrations induced on *Triticum aestivum* L. during seed germination and root elongation using different concentrations (50, 100, and 150 ppm) of commercial amino zinc nanoparticles (AZ NPs) [67]. Their results agree with the results reported in the current study, where it has been demonstrated that the incidence of aberrations in anaphase and telophase was 2–3 times higher in treated cells than in untreated cells. In another study, copper nanoparticles at concentrations of 18 g/mL, 36 g/mL, and 54 g/mL induced a broad spectrum of chromosomal abnormality in the root meristems of buckwheat (*Fagopyrum esculentum* Moench) [68].

Moreover, it has been reported that multinucleoli were the most common aberration recorded. That may be due to the accumulation of newly generated ribosomal RNA precursors that speed up the protein synthesis machinery, which may produce several nucleoli and account for the vigor expansion observed. Also, Heterochromatinization was observed following the Fe/Cu/P nanocomposite treatment. That may result from the transcriptional activity and cell proliferation, indicating that Fe/Cu/P nanocomposite may interfere with spindle fibers and enhance transcriptional activity, necessitating more ribosomes for increased protein synthesis, as observed by multinucleate cells. Otherwise, the nanoparticles could interfere and form complexes with nucleic acids and nuclear proteins, resulting in genetic data corruption and/or impaired DNA repair activities [69].

## Conclusion

In conclusion, a one-step synthesis process has been effectively employed to synthesize spherical Fe/Cu/P nanocomposite with a mean particle size of 4.35 ± 1.524 nm. Cu^2+^, Fe^2+^, and P^4+^ were detected in the dried nanocomposite at 14.533 ± 0.176, 5.200 ± 0.208, and 34.167 ± 0.203 mg/ml, respectively. The investigated Fe/Cu/P nanocomposite has exerted antibacterial activity against *E. coli* and *S. typhi*. It yielded a significant inhibition percentage against *Rhizoctonia solani* and good inhibition against *F. oxysporum*, reducing its germination rate and efficiency. The Fe/Cu/P nanocomposite was found to exert an antisnail effect against *L. carinatus* with a 76 ppm LC_50_ value. Wheat plants treated with this nanocomposite showed enhanced growth parameters and mitigated drought stress with improved mitotic index and chromosomal aberrations percent.Therefore, the current findings could be employed in securing food resources to meet the global food demand and provide a potential nanocomposite to be applied as an antimicrobial, antisnail and growth promoting agent in agricultural applications.

## Data Availability

All data generated or analyzed during this study are available from the corresponding author upon reasonable request.

## Abbreviations

ATCC: American Type Culture Collection
CA: Chromosomal aberration
CMC: Carboxy methyl cellulose
DNA: Deoxyribonucleic acid
FT‑IR: Fourier‑transform infrared
HR-TEM: High resolution- Transmission electron microscope
ICP-OES: Inductively coupled Plasma – Optical Emission Spectroscopy
LC_50_: Lethal concentration 50
MI: Mitotic index
NC: Nanocomposite
NPs: Nanoparticles
PDA: Potato dextrose agar
PIM: Percent inhibition of mycelial growth
ppm: Part per million
RNA: Ribonucleic acid
ROS: Reactive oxygen species
rpm: Round per minute
TEM: Transmission electron microscopy
XPS: X-ray Photoelectron Spectra
XRD: X-ray diffraction
